# Genome-wide association studies highlight novel risk loci for septal defects and left-sided congenital heart defects

**DOI:** 10.1186/s12864-024-10172-x

**Published:** 2024-03-07

**Authors:** Martin Broberg, Minna Ampuja, Samuel Jones, Tiina Ojala, Otto Rahkonen, Riikka Kivelä, James Priest, FinnGen, Aarno Palotie, Hanna M. Ollila, Emmi Helle

**Affiliations:** 1 Stem Cells and Metabolism Research Program, Faculty of Medicine, University of Helsinki, 00014 Helsinki, Finland; 2 Institute for Molecular Medicine Finland (FIMM), HiLIFE, University of Helsinki, 00014 Helsinki, Finland; 3 Department of Pediatric Cardiology, New Children’s Hospital, Pediatric Research Center, Helsinki University Hospital, 00029 Helsinki, Finland; 4 Wihuri Research Institute, 00290 Helsinki, Finland; 5 Faculty of Sport and Health Sciences, University of Jyväskylä, 40014 Jyväskylä, Finland; 6 School of Medicine, Stanford University, Stanford University, Stanford, CA 94305 USA; 7 Center for Genomic Medicine, Massachusetts General Hospital, Boston, MA USA; 8 Program in Medical and Population Genetics, Broad Institute, Cambridge, MA 02142 USA; 9 Department of Anesthesia, Critical Care and Pain Medicine, Massachusetts General Hospital and Harvard Medical School, Boston, MA 02114 USA; 10 Haartmaninkatu 8, Helsinki, 00014 Finland; 11 Population Health Unit, Finnish Institute for Health and Welfare, Helsinki, 00271 Finland

**Keywords:** Genetics, Congenital heart disease, Genome-wide association study, Single nucleotide polymorphisms

## Abstract

**Background:**

Congenital heart defects (CHD) are structural defects of the heart affecting approximately 1% of newborns. They exhibit low penetrance and non-Mendelian patterns of inheritance as varied and complex traits. While genetic factors are known to play an important role in the development of CHD, the specific genetics remain unknown for the majority of patients. To elucidate the underlying genetic risk, we performed a genome wide association study (GWAS) of CHDs in general and specific CHD subgroups using the FinnGen Release 10 (R10) (*N* > 393,000), followed by functional fine-mapping through eQTL and co-localization analyses using the GTEx database.

**Results:**

We discovered three genome-wide significant loci associated with general CHD. Two of them were located in chromosome 17: 17q21.32 (rs2316327, intronic: *LRRC37A2*, Odds ratio (OR) [95% Confidence Interval (CI)] = 1.17[1.12–1.23], *p* = 1.5 × 10^–9^) and 17q25.3 (rs1293973611, nearest: *BAHCC1*, OR[95%CI] = 4.48[2.80–7.17], *p* = 7.0 × 10^–10^), respectively, and in addition to general CHD, the rs1293973611 locus was associated with the septal defect subtype. The third locus was in band 1p21.2 (rs35046143, nearest: *PALMD*, OR[95%CI] = 1.15[1.09–1.21], *p* = 7.1 × 10^–9^), and it was associated with general CHD and left-sided lesions. In the subgroup analysis, two additional loci were associated with septal defects (rs75230966 and rs6824295), and one with left-sided lesions (rs1305393195). In the eQTL analysis the variants rs2316327 (general CHD), and rs75230966 (septal defects) both located in 17q21.32 (with a LD r2 of 0.41) were both predicted to significantly associate with the expression of *WNT9B* in the atrial appendage tissue category. This effect was further confirmed by co-localization analysis, which also implicated *WNT3* expression in the atrial appendage. A meta-analysis of general CHD together with the UK Biobank (combined *N* = 881,678) provided a different genome-wide significant locus in *LRRC37A2*; rs16941382 (OR[95%CI] = 1.15[1.11–1.20], *p* = 1.5 × 10^–9^) which is in significant LD with rs2316327.

**Conclusions:**

Our results of general CHD and different CHD subcategories identified a complex risk locus on chromosome 17 near *BAHCC1* and *LRRC37A2*, interacting with the genes *WNT9B*, *WNT3* and *MYL4*, may constitute potential novel CHD risk associated loci, warranting future experimental tests to determine their role.

**Supplementary Information:**

The online version contains supplementary material available at 10.1186/s12864-024-10172-x.

## Background

Congenital heart defects (CHD) are globally the most common birth defect. CHD include a wide range of structural malformations of the heart and great vessels ranging from mild defects, such as bicuspid aortic valve (BAV), which might not cause any symptoms, to critical defects, such as transposition of the great arteries (TGA) and hypoplastic left heart syndrome (HLHS), which are generally lethal without surgical interventions. CHD have their origin in the first trimester of pregnancy [1, 2], and may occur as an element of a syndrome or as an isolated defect. Although monogenic cases have been reported, most isolated CHD are thought to be complex traits with reduced penetrance and variable disease severity and phenotype [3]. In addition to genetic factors, environmental exposures such as maternal obesity, diabetes, and certain medications increase the risk for CHD in the offspring [4–7].

Next-generation sequencing studies have identified that the haploinsufficiency of NOTCH*-* and VEGF-signaling pathway genes associate with left ventricular outflow tract obstruction (LVOTO) defects and tetralogy of Fallot (TOF), respectively [8–10]. Damaging variants in transcription factors, such as *NKX2-5, GATA4,* and *TBX5* have been associated with various CHD, and familial septal defects in particular [11–13]. Excess of rare inherited variants have been identified in isolated CHD subjects [14, 15]. In most isolated CHD cases, however, the genetic etiology remains to be identified.

As genome-wide sequence data are available for large populations, genome-wide association studies (GWAS) have been used as an additional tool to identify genetic determinants of CHD [16, 17]. Previous studies have identified genome-wide significant loci for all CHD [18] as well as for certain CHD subgroups, such as TOF [19], conotruncal heart defects and LVOTO [20], and coarctation of the aorta [21]. As genetic and phenotypic data for hundreds of thousands of individuals have become integrated into large biobank-based projects such as FinnGen, UK Biobank (UKBB), and Japan Biobank, the statistical strength for population-wide GWAS has increased (https://finngen.gitbook.io/documentation/). Here, we conduct a GWAS of general CHD and three CHD subcategories and identify three significant risk loci in the FinnGen and UK Biobank data.

## Results

### FinnGen GWAS highlights SNPs in chromosome 17 near *BAHCC1* and *LRRC37A2,* and in chromosome 1 near *PALMD* as significantly associated with CHDs

**Fig. 1:**
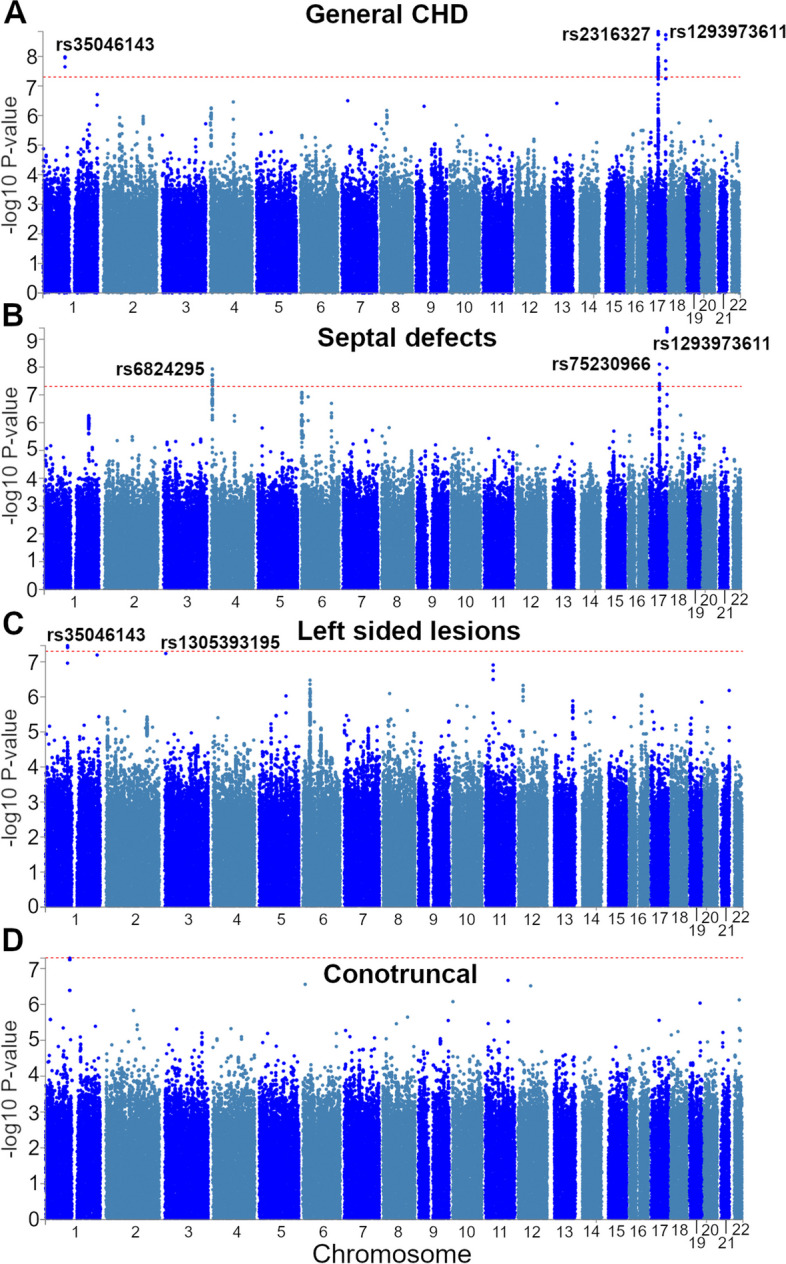
Lead SNPs associating with CHD identified in FinnGen R10. Manhattan plots of the different CHD categories: **A** general CHD, **B** septal defects, **C** left-sided lesions and **D** conotruncal defects, with genome-wide significant lead SNPs indicated, meaning the SNPs within a region with the highest association signal, as the other SNPs may only show association due to being in linkage disequilibrium (LD) with the lead signal

**Fig. 2:**
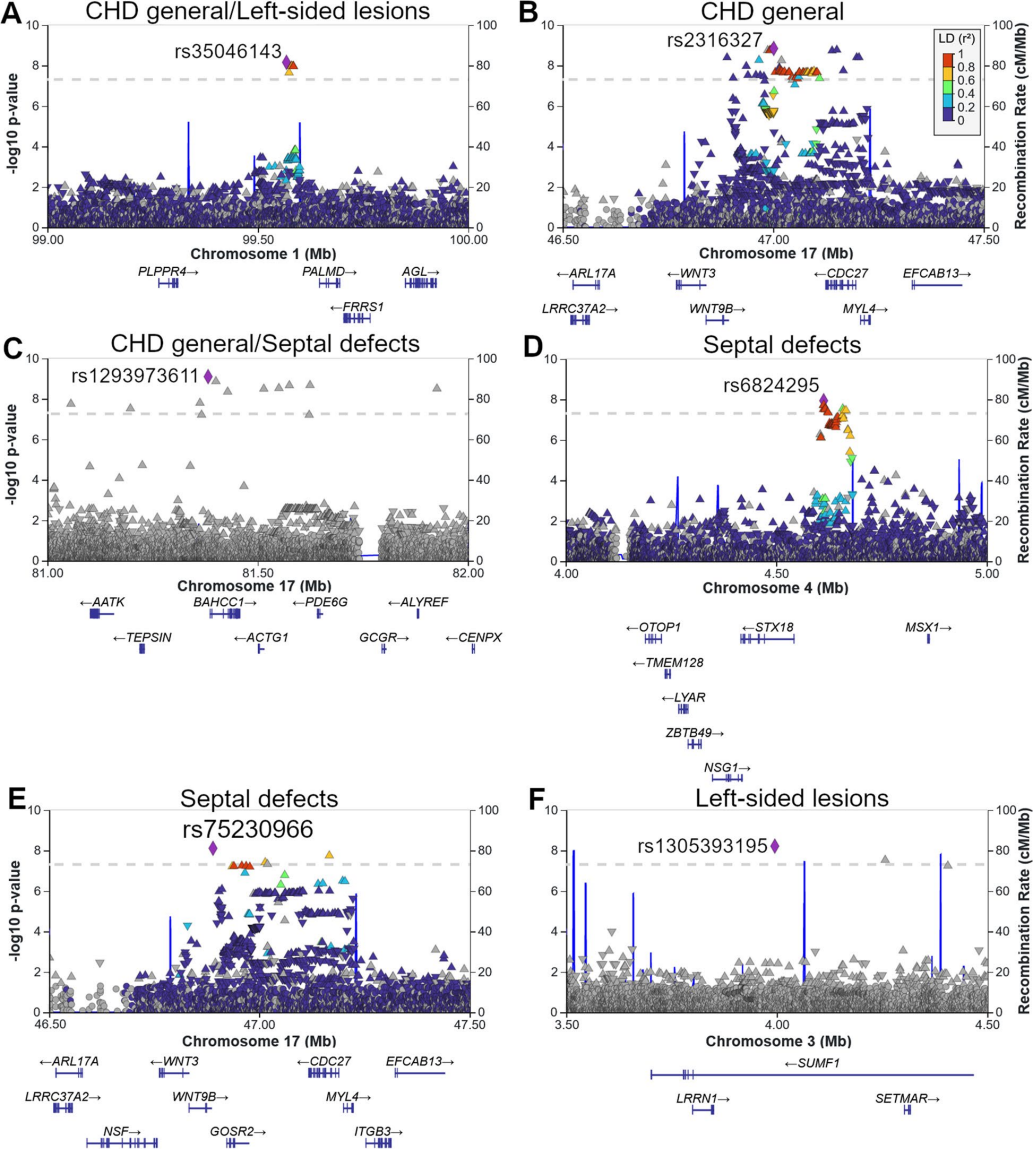
The lead SNPs are located on chromosomes 1, 3, 4 and 17. LocusZoom plots of **A** rs35046143 (general CHD and left-sided lesions), **B** rs2316327 (general CHD), **C** rs1293973611 (general CHD and septal defects), **D** rs6824295 (septal defects), **E** rs75230966 (septal defects), **F** rs1305393195 (left-sided lesions) and their nearby genes and SNPs. The LD indicated by colors in the plots was computed using the 1000 genomes project as reference panel [22]

We detected three loci in the general CHD GWAS (Table 1) rs35046143 (chr1, near *PALMD*, bp 99,567,756), rs2316327 (chr17, intronic *LRRC37A2*, bp 47,001,132) and rs1293973611 (chr17, near *BAHCC1*, bp 81,382,139, FinnGen imputed INFO score 0.97) as depicted in Fig. 1 and Table 1. The SNP rs1293973611 was also found significant in a GWAS of septal defects (Odds ratio (OR) [95% Confidence Interval (CI)] = 6.23[3.60–10.80], *p* = 1.2 × 10^–10^). Furthermore, SNPs rs75230966 (chr 17, intronic *LRRC37A2*, bp 46,890,164) and rs6824295 (chr 4, near *STX18*, bp 4,612,553) also associated with septal defects. Both rs2316327 and rs75230966 are found in the chromosomal band 17q21.32 (Fig. 2). In FinnGen the calculated LD r^2^ between two SNPs is 0.41. In analyzing left-sided lesions, the general CHD associated SNP rs35046143 in chr1 was significant, and an additional SNP rs1305393195 (chr 3, near *PNTP1P1*, bp 3,995,049, FinnGen imputed INFO score 0.93) was found to be genome-wide significant (*p* = 6.2 × 10^–9^, Table 1). These signals disappeared when we ran a sensitivity test using only cases with aortic valve disease under 50 years of age (results not shown). The overall quantile–quantile *p* distribution plot showed no evidence of inflation (Supplemental Fig. 1). No genome-wide significant associations were found for conotruncal defects. As a sensitivity analysis we performed a GWAS for general CHD but removing patients with Atrial Septal Defects (ASD) (ICD-10 codes: Q21.10, Q21.11), and lost all significant signals.

**Table 1:** Lead SNPs detected in FinnGen. Specific data for all lead SNPs across the three CHD categories with significant findings. Conotruncal defects results are not included as they did not include any genome-wide significant SNPs. NFEE signifies our results compared to Non-Finnish Non-Estonian Europeans, based on the GnomAD 2.1 reference panel containing 5421 individuals. MAF signifies minor allele frequency

Trait	Lead SNP	GRCh38 Position	Ref Allele	Alt Allele	Odds Ratio [95% Confidence Interval]	*p*	Finnish Enrichment vs NFEE in GnomAD 2.1	MAF
**General CHD**								
	rs35046143	1:99,567,756	TA	A	1.15[1.09–1.21]	7.1 × 10^–9^	NA GnomAD 2.1	0.48
	rs2316327	17:47,001,132	G	A	1.17[1.12–1.23]	1.5 × 10^–9^	1.6	0.29
	rs1293973611	17:81,382,139	C	T	4.48[2.80–7.17]	7.0 × 10^–10^	NA GnomAD 2.1	9.3 × 10^–4^
**Septal defects**								
	rs6824295	4:4,612,553	C	T	1.23[1.15–1.33]	1.2 × 10^–8^	1	0.24
	rs75230966	17:46,890,164	G	A	1.27[1.17–1.38]	7.9 × 10^–9^	1.3	0.16
	rs1293973611	17:81,382,139	C	T	6.23[3.60–10.80]	1.2 × 10^–10^	NA GnomAD 2.1	9.3 × 10^–4^
**Left-sided lesions**								
	rs35046143	1:99,567,756	TA	A	1.17[1.11–1.24]	3.2 × 10^–8^	NA GnomAD 2.1	0.48
	rs1305393195	3:3,995,049	T	G	11.02[10.15–11.97]	6.2 × 10^–9^	NA GnomAD 2.1	2.7 × 10^–4^

### Conditional analysis for rs2316327 and rs75230966

To assess if rs2316327 and rs75230966 represent independent risk variants, conditional analysis were conducted. The conditional analyses resulted in a *p*-value = 0.02 for rs2316327 for CHD general conditioned on rs75230966 as a covariate. Vice-versa, a *p*-value = 0.007 for rs75230966 for Septal defects when conditioned on rs2316327.

### CHD associated SNPs in 17q21.32 are associated with *WNT9B *and *WNT3* expression

To understand the possible functional consequences of the identified lead SNPs on gene expression we computed expression quantitative trait locus (eQTL) analysis focusing on heart tissues. We computed eQTLs for all lead SNPs with nearby genes ± 500kbp from the SNP position, using the GTEx data and discovered a potential effect by rs2316327 and the nearby rs75230966 on the expression of *WNT9B* (eQTL normalized effect size (NES) = 0.18 *p* = 2.8 × 10^–3^ and NES = 0.2 *p* = 1.2 × 10^–2^, respectively), in atrial appendage tissue. A co-localization analysis using LocusFocus [23], which uses a 1Mbp window for finding the co-localization of a SNP signal and eQTL data from GTEx V8. further demonstrated significant association for rs2316327 with *WNT3* (Simple Sum (SS) *p* = 5.1 × 10^–3^) and *WNT9B* (SS *p* = 3.9 × 10^–8^) in atrial appendage tissue. For rs75230966 the co-localization analysis in atrial appendage tissue demonstrated significant association with *WNT3* (SS *p* = 5.1 × 10^–3^) and *WNT9B* (SS *p* = 1.3 × 10^–6^). There were no significant GTEx and LocusFocus results for the other lead SNPs in this study.

### Meta-analysis with UKB supports association of 17q21.32 locus with CHD

We then examined the association of the lead SNPs with cardiac phenotypes in the UK Biobank data. While the lead variants did not associate in the UKB data, a meta-analysis of both FinnGen and UKB (total *N* = 881,678) for general CHD discovered an association at the same 17q21 locus with rs2316327 but a different lead variant rs16941382 (chromosome 17, bp 46,966,142, intronic in *LRRC37A2*) as genome-wide significant lead SNP (meta-analysis OR[95%CI] = 1.15[1.11–1.20], *p* = 1.5 × 10^–9^, Table 2). The variant rs16941382 demonstrates significant association with CHD in the FinnGen data (OR[95%CI] = 1.17[1.11–1.24], *p* = 5.5 × 10^–9^) and is in significant LD with rs2316327 (r^2^ = 0.67), thus likely reflecting the same signal. Furthermore, eQTL analysis and colocalization showed an association with *MYL4* expression: NES 0.1, *p* = 2.3 × 10^–3^, and *WNT9B*: NES 0.16, *p* = 1.3 × 10^–2^, both in atrial appendage in agreement with the FinnGen only results.

**Table 2:** Lead SNPs across cohorts. List of the general CHD lead SNP across the cohorts used in this study. NA signifies not available (SNP not present in summary statistics)**.** OR [95% CI] signifies Odds ratio [95% confidence interval]

	FinnGen		UKB		Meta-analysis	
SNP	OR [95% CI]	*p*	OR[95%CI]	*p*	OR[95%CI]	*p*
rs35046143	1.15[1.10–1.21]	7.1 × 10^–9^	NA	NA	NA	NA
rs2316327	1.17[1.12–1.23]	1.5 × 10^–9^	1.08[1.00–1.17]	6.0 × 10^–2^	1.14[1.09–1.20]	2.0 × 10^–9^
rs1293973611	4.48[2.80–7.17]	7.0 × 10^–10^	NA	NA	NA	NA
rs16941382	1.17[1.11–1.24]	5.5 × 10^–9^	1.11[1.02–1.19]	2.1 × 10^–2^	1.15[1.11–1.20]	1.5 × 10^–9^

### Phenome wide association testing in FinnGen suggest rs2316327, rs75230966, and rs35046143 are associated with a larger spectrum of cardiovascular endpoints

The FinnGen dataset includes longitudinal register data recording phenotypes and health events (including ICD-based diagnosis) of the study subjects obtained from Finnish national registers including data on all births and hospital care in Finland. Specifically, the Medical Birth Register includes data of all births in Finland since 1987 and the Care Register for Health Care (formerly Hospital Discharge Register) includes diagnosis and other data parameters of all hospital care in Finland since 1969. These registers are maintained by the Finnish Institute for Health and Welfare (THL) and have been shown to be reliable [24, 25]. This enables Phenome Wide Association Testing (PheWAS) to examine a single significant variant horizontally across other diseases and precomputed phenotypes to understand which other conditions are associated with the variant of interest. In Supplemental Fig. 2 we demonstrate the overall significant associations of the lead SNPs rs2316327 and rs75230966 with other binary cardiovascular endpoints in FinnGen similarly made using REGENIE. These endpoints include hypertension (cases = 122,996 controls = 289,117, *p* = 9.5 × 10^–9^ and 3.9 × 10^–18^ respectively), use of antihypertensive medication (cases = 222,561 controls = 189,620, *p* = 8.3 × 10^–14^ and 9.9 × 10^–21^ respectively), atrial fibrillation and flutter (cases = 50,743 controls = 210,652, *p* = 1.1 × 10^–9^ and 2.3 × 10^–17^ respectively) and cardiac arrhythmias (cases = 74,000 controls = 239,778, *p* = 4.9 × 10^–9^ and 0.5 × 10^–11^ respectively). The SNP rs35046143 that associated with left-sided lesions associated also with non-rheumatic valve disease (cases = 22,653 controls = 312,154, *p* = 3.7 × 10^–24^), valvular heart disease excluding rheumatic fever (cases = 22,818 controls = 312,154, *p* = 7.2 × 10^–24^), and operated calcific aortic valvular stenosis (cases = 9,870 controls = 402,311, *p* = 1.7 × 10^–26^).

## Discussion

Here we report the results of a GWAS of CHD traits in the FinnGen R10 database, where we detected three main regions associated with CHDs. Our aim was to elucidate the genetics behind CHD in general, but also explore specific sub-categories; conotruncal defects, septal defects and left-sided lesions. We discovered two genetic loci with the general CHD category GWAS in chromosome 17 (bands 17q21.32 and 17q25.3) and one in chromosome 1 (1p21.2). In addition, using GTEx we show that the loci are related to WNT9B and WNT3 expression levels in the heart. Overall, our findings elucidate the genetic etiology behind CHD complementing the growing body of literature of these relatively poorly understood severe diseases. We focus in this study on the lead variants for associations, to avoid reporting potential false positives. Of the lead variants described, rs1305393195 had the lowest MAF at 2.7 × 10^–4^, along with rs1293973611 having a MAF of 9.3 × 10^–4^ This means these two SNPs should have a population-wide genotype count of over 106, but both are rare variants specific to the Finnish population. As neither of these two SNPs demonstrated any significant eQTLs nor neighboring SNPs with LD r2 above 0.71, their association to their respective CHDs remains unclear. We did not discover any genome-wide significant lead SNPs for conotruncal defects.

The loci with the lead SNPs in chromosome 17 was also detected as significant in an analysis of the septal defect subcategory, while SNPs in the band 1p21.2 were found to significantly associate with left-sided lesions. The region in chromosome 17 (band 17q21.32), where the SNP rs2316327 associated with general CHD and rs75230966 and associated with septal defects are located, contains several genes linked to heart development, CHD, and other cardiac phenotypes; *MYL4, CDC27, GOSR2* and *WNT9B* [18, 26–28]. A recent GWAS study on patients with anomalies of thoracic arteries and veins identified three significant SNPs within the *GOSR2* locus in chromosome 17 close to rs11570508 [18]. Furthermore, the rs17608766 variant in *GOSR2* has been associated with a reduced aortic valve area [29] indicating importance of this locus in cardiac development. The same study indicated a that the risk variants in the 17q21.32 influence the expression of *WNT3,* which is in accordance with our findings, and demonstrated that *WNT3* is expressed in cardiac progenitor cells [18].

According to eQTL and co-localization follow-up analyses of the genome-wide significant SNPs from FinnGen and a meta-analysis together with UKB (rs2316327, rs7523096 and rs16941382), we detected effects on primarily *WNT9B*. The SNP rs2316327 demonstrated an eQTL NES = 0.18, *p* = 2.8 × 10^–3^ for *WNT9B*. Wnt signaling is required for second heart field development, and *Wnt9b* has shown to have an important role in endocardial endothelial cushion development in the developing heart in mice [26, 30]. Animal studies modeling cardiac development in mouse and zebrafish show that *Wnt9b* is expressed specifically in endocardial endothelial cells overlying the developing atrioventricular canal and outflow tract [31], and it has been suggested that congenital heart defects may arise due to defects in the Klf2-Wnt9b mechanotransduction pathway [26]. Thus, these data could suggest a pathogenic mechanism for our finding. Overall, the positive NES eQTL data of rs2316327, rs7523096 and rs16941382 on the expression of the *WNT9B* and significant co-localization results for these, suggests a positive effect of the gene on development of CHD in heart tissue. However, this connection will need to be tested experimentally. It is important to note that none of the eQTL multiple test corrections yielded significance.

The GTEx analysis of the SNP rs16941382 identified in the meta-analysis of FinnGen and UKB indicated significance for *MYL4* expression*. MYL4* encodes the atrial light chain-1 (ALC-1) protein, which is expressed in the human heart during development [32]. After birth, the expression decreases in the ventricles but remains in the atria [33]. ALC-1 has a role in sarcomere assembly and fine tuning of cardiac contractility. The expression of *MYL4* has shown to abnormally persist in ventricular tissue of individuals with CHD [34], and the ventricular analog *MYL3* is replaced by re-expression of *MYL4* in failing and hypertrophied hearts, resembling a fetal remodeling pattern associated with ventricular dysfunction [33, 35]. *MYL4* loss-of-function variants have been associated with atrial cardiomyopathy and atrial arrhythmias such as atrial fibrillation (AF) [27, 36–38], demonstrating that although previously not associated with CHD directly, its role in cardiac development and function is important, and thus, could be a plausible candidate gene for CHD. Furthermore, we used the lead SNP positions in the Human Heart Genome Browser to search the ATACseq data for peaks indicating regulatory motifs [39], but no significant peaks were found.

Interestingly, in addition to CHD, the SNPs rs2316327 and rs75230966 were associated with hypertension, use of antihypertensive medications, atrial fibrillation and flutter, and other arrhythmias. Children and adults with CHD have increased risk for atrial fibrillation and flutter [40, 41], and adults with CHD have increased cardiovascular morbidity relative to the general population [42]. Although anatomical factors such as anomalous vessel anatomy and abnormal atrial hemodynamics, and disease related conditions in the heart such as progressive valvulopathy, residual shunts, and atrial scars from previous heart surgery are likely to be important predisposes for atrial arrhythmias and other cardiovascular morbidity in CHD, it is tempting to speculate that shared genetic factors could potentially increase the risk for both developing CHD and later cardiovascular morbidity. In fact, atrial arrythmias are a common phenotype in Holt-Oram syndrome, a developmental disorder that leads to heart and limb malformations. Holt-Oram syndrome is partially caused by variants in the *TBX5* gene [43, 44], and GWAS studies on AF have shown several significant loci with nearest genes known to be important for heart development and causal for CHD, such as *NKX2-5, GATA4, GJA1,* and *GJA5* [11, 45]. It has been also shown that women whose infants have congenital heart defects have an increased risk and earlier disease onset for cardiovascular morbidity, including atherosclerotic cardiovascular disease, ischemic heart disease, hospitalization due to cardiac diseases and cardiac transplantation [46]. Thus, these associations could indicate shared genetic risks.

The lead SNP findings did not replicate in the UKB data, but a nearby SNP showed genome-wide significance in the meta-analysis. This could be due to differences in the number and severity of included CHD cases between the two CHD cohorts as depicted in Supplemental Table 1. In addition, it is possible that the definitions varied somewhat, as different coding systems were used. The FinnGen samples have been gathered from Finnish biobanks including samples collected from hospitalized patients of all ages, whereas UKB samples are in average from older individuals. Indeed, there was a higher percentage of affected individuals as well as more severe phenotypes among the FinnGen individuals. Another explanation could be the different genetic backgrounds of these two cohorts.

Our approach has some limitations. The discovery of novel loci in GWAS studies does not prove functional causality, and validation studies should be conducted. In addition, ICD10-based studies are limited by the fact that mild CHD such as BAV and hemodynamically insignificant ASD are often underdiagnosed as they may be asymptomatic. However, removing the ASD patients (1215 individuals) from our study diminished the significant association signals, which is understandable since it meant a significant loss of power (a third) from the cases. Additionally, the conditional analyses identified the general CHD loci as independent from the septal defect loci. The loci on chromosome 1 was not identified as significant in the septal defect GWAS, further demonstrating that the results of the general CHD GWAS were not entirely dependent on septal defects such as ASD. As the region includes several genes linked to heart development, and SNPs in this region has been associated with anomalies of thoracic arteries and veins [18] and reduced aortic valve area [29], it is likely that this locus has importance for cardiac development in general. Previous studies report that approximately 0.8–1.0 percent of the population are affected by CHD [47, 48], and of the FinnGen R10 individuals, 0.8% have at least one CHD diagnosis. While it is possible that some controls in FinnGen are affected with CHD, the registers where the diagnoses were acquired from have been validated and are concluded to be reliable [24], thus the case status can be anticipated to be defined correctly. Finally, our association results are from the isolated Finnish population, which is genetically unique. On the other hand, our study highlights the potential of FinnGen to identify disease associated high-impact variants that are very rare or absent in other populations.

## Conclusions

In summary, our analysis of different CHD categories primarily using FinnGen identifies a complex risk locus for CHD on chromosome 17 for CHD in general and septal defects. This locus is associated with *WNT3*, *WNT9B* and *MYL4*. However, further experimental and clinical data is needed to determine the impact and roles of these genes on CHD onset. Additional data would be needed to establish the independencies and impact of the identified lead SNPs rs2316327 and rs75230966 on the onset of CHDs. These may be two independent contributing signals within a locus, or neither capturing the true loci, as the conditional analyses identified both as independent signals but both affecting the significance of each other. The associations between the identified loci and other cardiovascular conditions, such as AF and flutter and hypertension may suggest shared genetic etiology in developmental defects and adult cardiovascular morbidity.

## Methods

### Study cohorts and phenotypes

#### FinnGen

FinnGen is a large-scale research project that aims to genotype 500,000 Finnish participants recruited from hospitals as well as prospective and retrospective epidemiological and disease-based cohorts. The participants are of Finnish ancestry. These data are combined with longitudinal registries that record phenotypes and health events (including ICD-based diagnosis) over the entire lifespan including the Care Register for Health Care (inpatient and outpatient), Causes of Death Registry, the National Infectious Diseases Registry, Cancer Registry, Primary Health Care Registry (outpatient) and Medication Reimbursement Registry. This study used data from FinnGen R10, which consists of > 390,000 individuals (see next paragraph for specific numbers in each CHD category that was analyzed). FinnGen utilizes custom ThermoFisher Axiom microarrays for capturing variants, and individuals are then imputed against a whole-genome sequenced Finnish reference panel [49] using IMPUTE2 version 2.3 [50]. Participants without Finnish ancestry based on principal component analysis (PCA) against 1000 genomes reference samples have been removed from the FinnGen study [49]. Approximately 73.6% of participants have a 3rd-degree relative or closer among the cohort. Furthermore, genetic duplicates, monozygotic twins (one of the pair kept) and other genetic outliers have been removed. At the variant level, FinnGen QC removes variants with high missingness (> 2%), low Hardy–Weinberg equilibrium (*P* < 1 × 10^–6^) and minor allele count < 3. For determining ancestry, the FinnGen PCA analysis was performed on unrelated individuals within FinnGen (relationship is more than a 3rd degree relative) includes the following variant filtering: exclusion of variants with info score < 0.95, exclusion of variants with missingness > 0.02 (based on the GP; see conversion), exclusion of variants with MAF < 0.05 and LD pruning with window interval of 500 kb and 50 kb steps, and an r^2^ filter of 0.1 (https://finngen.gitbook.io/documentation/methods/phewas/quality-checks) [49].

### CHD subjects in FinnGen

In the FinnGen research project, the CHD categories used for this study consisted of general CHD (3506 cases, 392,436 controls), conotruncal defects (404 cases, 392,942), left-sided lesions (LVOTO and early aortic valve disease diagnosed under 50 years of age; 2382 cases, 392,503 controls) and septal defects (1955 cases, 392,428 controls) using FinnGen R10. General CHD and the CHD subcategories were defined according to ICD-10 codes as depicted in Supplemental Table 1. We used 50 years of age for early aortic valve disease as this is considered to be primarily associated with congenital heart defects, not deterioration over age [51]. We code variants in the X chromosome as 0,1,2 in females and 0,2 in males to account for the difference in number of X chromosomes in females and males.

From the FinnGen population we identified patients with a CHD diagnosis and from those excluded individuals with a diagnosis for syndromes that are known to include CHD phenotypes.

### REGENIE GWAS analysis

The GWAS for CHD was performed using REGENIE on the FinnGen R10 cohort using Finnish participants where over 70% had at least one 3rd degree relative or closer within the cohort [49, 52]. We utilized REGENIE to compute association statistics. To account for population stratification and relatedness, Regenie directly estimates the polygenic effects parameter β by using ridge regression, which corresponds to fitting a linear regression model with a L2 penalty to impose shrinkage [52]. The covariates used for the analysis included: sex, imputed age, age at death or end of follow up, 10 principal components and microarray genotyping batch. The default Regenie filtering of a minimum allele count > 5 was used, along with the firth regression option. A total of 16,960,158 SNPs across the autosomal and X chromosomes were included in the analyses.

We performed a validation GWAS of general CHD using UKB (cases = 2165, controls = 484 865). As UKB uses a slightly different ICD10 coding structure, we had to modify the codes used for defining general CHD, as well as utilize the General Practitioner Read Codes V2 and V3 (Supplemental Table 1). We performed the GWAS using the REGENIE software [52], correcting for sex, age, PC1-10, array used for SNP detection and assessment center. Additionally, we performed a GWAS meta-analysis using METAL (sample size based analysis) [53], combining the two GWAS datasets for a combined *N* = 881,678 (Table 3) and only considering SNPs that occur in both studies.

**Table 3:** Cohorts used for GWAS in this study

Cohort	Cases	Controls
FinnGen – General CHD	3506	392,436
UKB – General CHD	2156	484,865
Meta-analysis – General CHD	5662	877,301
FinnGen – conotruncal defects	404	392,942
FinnGen – left-sided lesions	2382	392,503
FinnGen – septal defects	1955	392,428

### Calculating eQTL data and co-localization

To test whether the genome-wide significant SNPs from the FinnGen GWAS could have an effect on the expression of nearby genes, we utilized the eQTL calculator function of the GTEx portal database (www.gtexportal.org) on all lead SNPs in this study [54, 55]. We examined expression in the following tissues: ‘heart—atrial appendage’ and ‘heart – left ventricle’. To further test the GTEx result for the SNPs demonstrating significant (*p* < 0.05) eQTL, we used LocusFocus [23] to calculate Simple Sum colocalization *p* for the lead SNPs in heart tissues using GTEx V8.

### Defining endpoint associations using PheWeb

In addition to CHD, FinnGen contains diagnosis codes and precomputed phenotypes. Therefore, instead of looking at each disease or GWAS alone, it is possible to examine a single significant variant horizontally across other diseases to understand which other diseases are associated with this variant. The FinnGen results website uses the PheWeb design interface for displaying SNP-endpoint associations for a convenient overview of GWAS results [56]. This approach was used to identify other endpoints associated with the lead SNPs. The FinnGen R10 data will become available to the public similarly as previous releases.

### Conditional analysis

We used the GCTA COJO software v1.94 [57] to detect independent signal selection (–cojo-slct) for each dataset (CHD general and Septal defects), where the respective lead SNPs were identified as independent signals. A conditional analysis (–cojo-cond) where we tested for both CHD general and Septal defects removed the respective lead SNP from the other.

### Supplementary Information

**Supplementary Material 1. **

**Supplementary Material 2. **

**Supplementary Material 3. **

**Supplementary Material 4. **

## Data Availability

The datasets used and/or analysed during the current study are available from the corresponding author on reasonable request.
