# Supplementary figures and images for: Genome-wide association studies highlight novel risk loci for septal defects and left-sided congenital heart defects
Source: BMC Genomics. 2024 Mar 7;25:256. doi: 10.1186/s12864-024-10172-x (PMC10918883; doi:10.1186/s12864-024-10172-x)

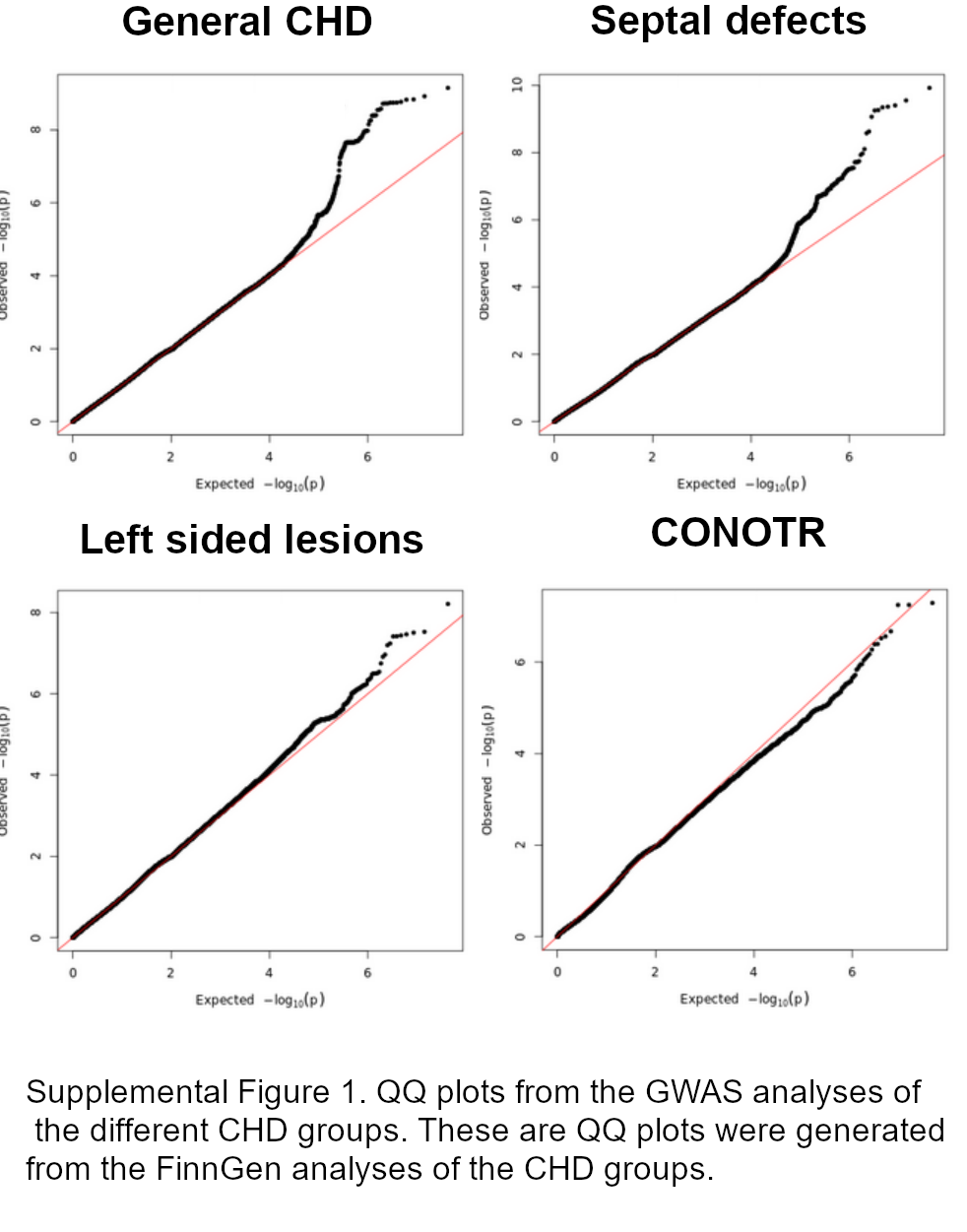

Supplement: Supplementary file 2 — Supplementary Material 2. [file 12864_2024_10172_MOESM2_ESM.tiff]

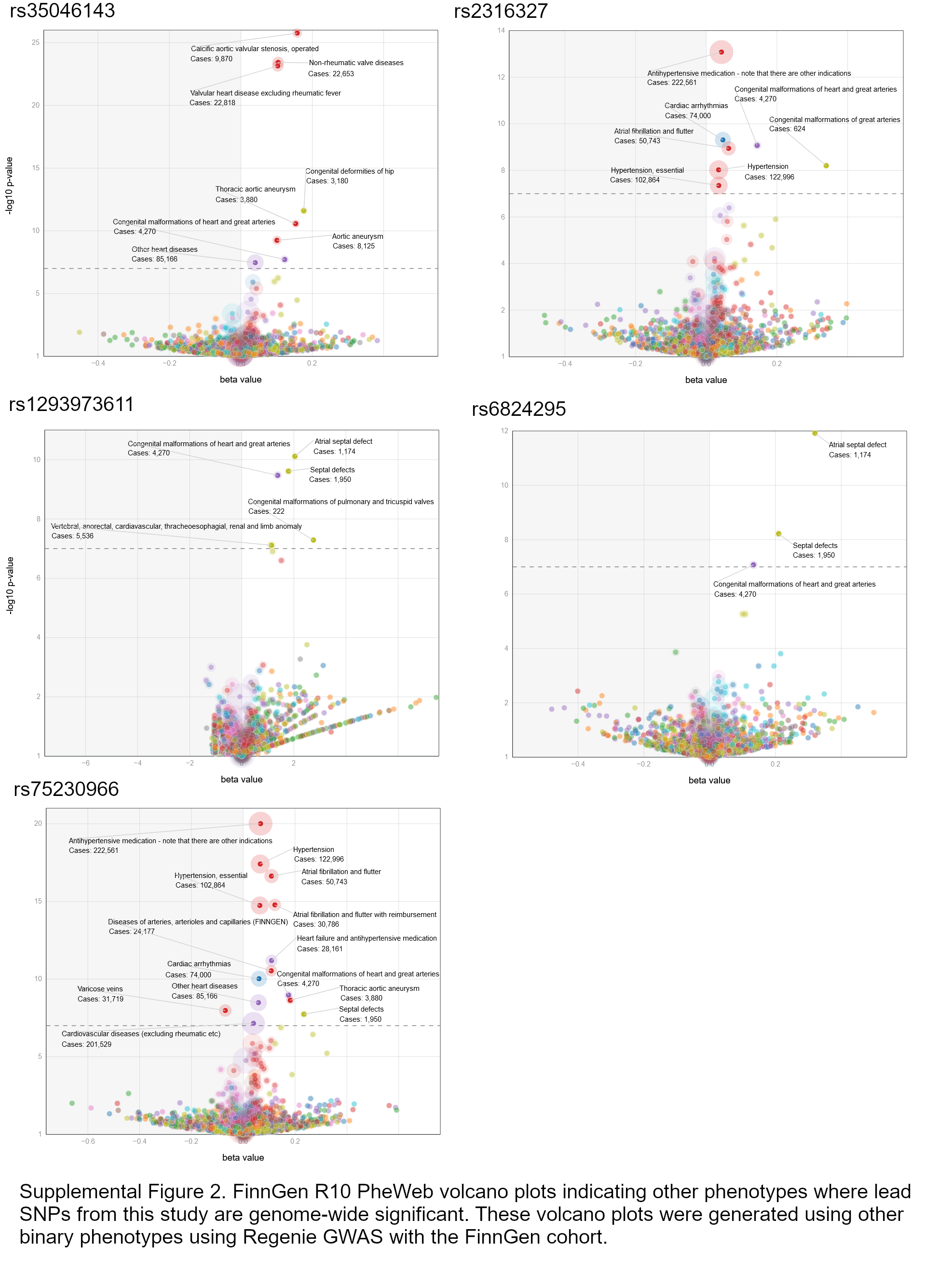

Supplement: Supplementary file 3 — Supplementary Material 3. [file 12864_2024_10172_MOESM3_ESM.tiff]
